# You and me versus the rest of the world: the effects of affiliative motivation and ingroup partner status on social tuning

**DOI:** 10.3389/fpsyg.2023.1060166

**Published:** 2023-08-17

**Authors:** Jeanine Lee McHugh Skorinko, Melissa-Sue John, Aidan Doyle, Natalia Carvajal Erker, Matthew Figueroa, Jeffrey Harnois, Grace Gately, Sarah Spear, Satia Marotta, Casey McKenna, Lisa Rossi, Kenedi Heather, Tyler Jaskoviak, Daniel Vega, Avik Vimal, Mariam Kobeissi, Maia Selkow, Katherine Rondina, Karen Ho, Alisionna Iannacchione, Marisol Sanchez, Keely Heyer, Catherine Pittelli, Emily Bendremer

**Affiliations:** ^1^Psychological and Cognitiive Sciences Program, Worcester Polytechnic Institute, Worcester, MA, United States; ^2^Department of Psychology, Family, and Justice Studies, University of Saint Joseph, West Hartford, CA, United States; ^3^Department of Psychological Sciences, University of Connecticut, Storrs, CT, United States; ^4^Department of Psychology, American University, Washington, WA, United States; ^5^Cognitive Sciences Program, Department of Social Science and Policy Studies, Worcester Polytechnic Institute, Worcester, United Kingdom

**Keywords:** perceived similarity, ingroup identification, affiliative motivation, social tuning, shared reality, social consensus, implicit attitudes

## Abstract

Bandura argues that individuals are more likely to engage in social learning when they identify with a social model and when they are motivated or rewarded. Therefore, in the present work, we investigate how these two key factors, perceived similarity and affiliative motivation, influence the extent to which individuals engage in social tuning or align their views with an interaction partner—especially if their partner’s attitudes differ from the larger social group. Experiment 1 (170 participants) explored the role of perceived similarity through group membership when needing to work collaboratively with a collaboration partner whose climate change beliefs differed from a larger social group. Experiment 2 (115 participants) directly manipulated affiliative motivation (i.e., length of interaction time) along with perceived similarity (i.e., Greek Life membership) to explore if these factors influenced social tuning of drinking attitudes and behaviors. Experiments 3 (69 participants) and 4 (93 participants) replicated Experiment 2 and examined whether tuning occurred for explicit and implicit attitudes towards weight (negative views Experiment 3 and positive views Experiment 4). Results indicate that when individuals experience high affiliative motivation, they are more likely to engage in social tuning of explicit and implicit attitudes when their interaction partner belongs to their ingroup rather than their outgroup. These findings are consistent with the tenets of Social Learning Theory, Shared Reality Theory, and the affiliative social tuning hypothesis.

## Introduction

According to Albert Bandura (1969), individuals learn how to navigate their social worlds by imitating those whom they identify with (i.e., their social models) through a process known as social learning. In other words, social learning and the people who serve as social models are important conduits in transmitting essential information about social environments. Moreover, Bandura (1969) contends that identification with those who serve as social models, as well as motivation or rewards, increase the likelihood that social learning will occur. This is because these factors make the thoughts and behaviors of the social model more “determinative cues” and increase the likelihood that individuals will match their response with their social models (Bandura, 1969, p. 217). Based on this conceptualization, it can be argued that one of the most important elements of social learning is social interactions with others. Therefore, the current work examines social learning in social interactions using a social tuning framework. More specifically, the current work examines the roles that perceived identification (or similarity) of an interaction partner (e.g., ingroup or outgroup member) and the desire to get along with someone (i.e., affiliative motivation) play in the alignment of one’s attitudes with an interaction partner, or social tuning.

Shared Reality Theory, from which the social tuning framework stems, posits that successful social interactions rely on developing a mutual understanding, or shared reality, with an interaction partner (Hardin and Higgins, 1996; Hardin and Conley, 2001). One reason that individuals may be motivated to experience shared reality is because this mutual understanding limits awkward social interactions. In other words, interaction partners, under the right conditions, may unconsciously align their views with an interaction partner—or social tune. Past research demonstrates that social tuning, like social learning, facilitates the transmission of beliefs and knowledge (Higgins and Rholes, 1978; Echterhoff et al., 2005; Sinclair and Lun, 2006; Weisbuch et al., 2009).

One factor that might facilitate social tuning is affiliative motivation, or the desire to get along with an interaction partner. More specifically, the affiliative social tuning hypothesis (Sinclair et al., 2005a,b) predicts that higher levels of affiliative motivation should increase the likelihood of engaging in social tuning to meet the goals of developing shared reality. Research corroborates that affiliative motivation leads to social tuning as those with higher affiliative motivation were more likely to tune towards an interaction partner than those with lower affiliative motivation (Sinclair et al., 2005a,b). Affiliative motivation increased the tuning of automatic racial and gender attitudes, and it also increased the likelihood of self-stereotyping (Sinclair et al., 2005a,b). While Bandura’s (1969) work did not look at affiliative motivation in this same way, he argued that interpersonal motivations and rewards could be catalysts for social learning.

Bandura (1969) also argued that identification, or perceived similarity, with another person was a key factor in social learning, as he states: “under certain circumstances, modeling can also be significantly influenced by real or assumed similarity between the observer and the model” (p. 244). Research on perceived similarity and interpersonal relationships shows that individuals are more attracted to targets that are similar than dissimilar (Newcomb, 1956; Jones and Daugherty, 1959; Byrne, 1961; Byrne et al., 1966; Tsui and O'Reilly, 1989; Glaman et al., 1996; Chen and Kenrick, 2002). Additional research demonstrates that individuals will perceive similarity with another person based on a number of different shared (or perceived to be shared) characteristics, such as race, gender, college affiliation, hometown, hobby, or even similar dress (Tajfel et al., 1971; Tajfel and Turner, 1979; Taylor and Moriarty, 1987; Dovidio et al., 1995; Ashburn-Nardo et al., 2001; Zebrowitz et al., 2007). Research has also found that individuals are more likely to match expressions with ingroup members more than outgroup members (Blocker and McIntosh, 2017).

In addition, intergroup relations research demonstrates that individuals evaluate one’s own group more positively in relation to other groups resulting in ingroup favoritism or an ingroup bias (Aberson et al., 2000; Spears et al., 2001). The ramifications of perceived similarity of ingroup membership does not stop at positive evaluations of a group but extends to interpersonal relationships and prejudice as well. For instance, in employment situations, gender similarity increases the likelihood of building a relationship with one’s supervisor (Kammeyer-Mueller et al., 2011). Similarly, when individuals recategorize outgroup members as part of a larger superordinate group, then prejudice and discrimination towards this former outgroup dissipate (Gaertner et al., 1993; Gaertner and Dovidio, 2000). In addition, research also shows that ingroup consensus on racial attitudes exerted more influence on a person’s own racial attitudes than outgroup consensus (Stangor et al., 2001). Thus, identification through perceived similarity influences who we want to get along with, how we evaluate and treat others, and when are likely to adjust our own attitudes.

Applying this work to a social tuning perspective, this should translate into being more likely to engage in social tuning with someone who is perceived to be more similar, rather than dissimilar, because it will be easier to develop shared reality with someone who shares things in common than someone who does not. However, to date, perceived similarity of the interaction partner has received little attention in the social tuning literature. One study conducted had small groups of participants encounter either a White or Black experimenter in front of their classroom (Sinclair et al., 2005a). The experimenters wore a plain t-shirt (expressing no views) or an “Eracism” t-shirt (expressing egalitarian racial attitudes). Participants then completed a paper and pencil version of the Race Implicit Association Test (IAT; Greenwald et al., 1998, 2003). This study found that participants automatic racial attitudes were more associated with the shirt worn by the experimenter than the race of the experimenter. While these findings imply that perceived similarity through group membership might not influence social tuning, this experiment was run in small groups so it is unclear of whether the identities of the group members also played a role in the social tuning process or if the results would be different in a one-on-one interaction.

In addition, past work has not investigated the effects that social consensus has on the social tuning process. Past work by Stangor et al. (2001) found that learning about ingroup social consensus towards a racial group influenced participants own racial attitudes more than learning about outgroup social consensus. It is unclear what effect social consensus, especially ingroup social consensus, might have when interacting with a partner whose views are inconsistent with the ingroup social consensus.

Thus, in the current work, we investigate two key factors that are relevant to social learning: identification through perceived similarity and the motivation to get along with another person. More specifically, we are interested in the role perceived similarity and affiliative motivation play in the social tuning process. Given the role that ingroup social consensus can play in individuals own attitudes (Stangor et al., 2001), we also explore how ingroup social consensus influences in social tuning. We present four experiments that examine these factors. In each experiment, participants believe they will interact with an ostensible interaction partner. They also learn about their partner’s beliefs and how they differ from the beliefs of their larger social group (ingroup). In Experiment 1, we examine the role of perceived similarity on social tuning when needing to work collaboratively with a collaboration partner whose beliefs about climate change are different than a larger social group (e.g., social consensus). In Experiment 2, we directly manipulate the affiliative motivation that was held constant in Experiment 1 to better understand the role it plays along with perceived similarity on social tuning with an interaction partner whose views do not align with a larger social group. Experiments 3 and 4 extend this work by investigating whether affiliative motivation and perceived similarity also influence the social tuning of implicit attitudes, especially when the interaction partner’s beliefs are inconsistent with the larger social group.

## Experiment 1

This experiment investigated whether perceived similarity though group memberships influenced the extent to which individuals engaged in social tuning. We also explored the role that social consensus plays in the social tuning process based on past work that shows the ingroup social consensus can influence individual’s own beliefs (Stangor et al., 2001). In Experiment 1, we held affiliative motivation constant such that all participants believed they needed to work collaboratively with another person. Participants then learned that their ostensible interaction partner was either similar to them in group membership (i.e., participating through the same platform) or personal preferences (i.e., preferred same animal) or different. To examine the role of social consensus in social tuning, participants learned that their partner believed climate change was a more pressing issue than other participants in the collaborative portal. We predicted that participants would be more likely to engage in social tuning with their partner whose views differ from the larger social group when the partner is part of their ingroup rather than the outgroup. We predicted this because Shared Reality Theory (Hardin and Higgins, 1996; Hardin and Conley, 2001) contends that we seek to develop mutual understanding with an interaction partner to facilitate a smooth interpersonal interaction, and there is likely normative pressure to fit into one’s ingroup.

## Method

### Participants

One hundred and seventy individuals (107 males; 63 females) with the average age of 35 participated for a small monetary reward (Amazon’s Mechanical Turk) or for course credit and a chance to win a raffle prize (college participants). Eighty percent of the sample was White (138 White; 8 Black; 7 Asian; 7 Hispanic/Latinx; 1 Middle Eastern/North African; 7 Multi-Racial; 1 Other; 1 Unreported). All participants gave informed consent. Nine participants were not fully engaged in the experiment (e.g., did not complete it, completed it in less than 5 min, or wrote they did not care), and six participants did not believe they would collaborate with anyone. These participants were removed from the analyses. Therefore, the analyses are based on 155 participants (94 males; 127 White).

### Design and materials

To study the effects of group membership on social tuning, this experiment utilized a 2 (Partner Platform Membership: Same Platform or Different Platform) x 2 (Partner Animal Preference: Same Preference or Different Preference) between-participants design on attitudes towards climate change.

#### Affiliative motivation

We held affiliative motivation constant in this experiment by telling participants that we were piloting a new collaboration portal and that after doing some independent tasks they would meet their partner and complete a collaborative task in the portal and provide feedback on the collaborative portal.

#### Partner group membership manipulations

We manipulated perceived similarity with the partner in two different ways. Participants learned that their partner was either participating through the same or different platform (e.g., Amazon’s Mechanical Turk or their college participant pool). In creating their supposed profile for the collaboration portal, participants indicated whether they preferred cats or dogs. While reviewing their partner’s ostensible profile, they learned their partner preferred the same or different animal. Thus, we used similarities in group memberships to determine ingroup status and differences to determine outgroup status.

#### Perceived views of the partner and larger social group

To see if participants engaged in social tuning with their partner when their views differ from the larger social group, participants were led to believe that their partner believed climate change was an important issue and supported sustainable efforts. To do this, participants saw that their ostensible partner selected green leaves as their icon and read a short bio that said: “In my free time, I like to read. I think climate change is a really important topic today. I try to be ‘green’ and I volunteer with a local organization that promotes sustainable living!”

To show participants how their partner’s views compared to a larger social group, the supposed collaboration portal showed them a graphic depicting how their partner compared to others who had completed profiles in the system. Participants always learned that there were a relatively equal number of people from each platform and an equal number of people preferred each type of animal. However, when they saw the graphic about the hot topic preferences, it was clear that most people in the portal did not believe that climate change was as important as their partner did.

#### Social tuning measure

To measure social tuning, we measured participants’ self-reported climate change attitudes using the 15-item Climate Change Attitude Scale (1 = strongly disagree; 7 = strongly agree; Christensen and Knezek, 2015). Participants indicated their beliefs on climate change, such as: “I believe our climate is changing”; “The actions of individuals can make a positive difference in global climate change”; “We cannot do anything to stop global climate change.” Five items were reverse scored, and items were averaged together. Higher numbers indicate beliefs that climate change needed attention (see Appendix A for items and reverse scoring).

#### Follow-up and demographics

To increase believability that we were interested in different hot topics and not just climate change, participants also completed a questionnaire about the legalization of marijuana. We assessed their memory for the platform their partner was participating through, their partner’s animal preference, the hot topic that their partner thought was important, and inquired into their thoughts how the interaction would go. We also collected basic demographic information, such as gender and ethnicity, and asked participants about any suspicions they had while taking the experiment.

### Procedure

Participants believed they were participating in a study piloting a new collaboration portal and that we were seeking feedback on the portal. Participants learned they would provide information about themselves to create their profile and then the portal would randomly match them with another participant, and they would get to see their partner’s profile. To create this supposed profile, participants indicated whether they were participating through Amazon’s MTurk or their college’s participant pool. Participants also specified if they preferred cats or dogs and wrote a short bio to share with their partner. Participants than indicated from a list of seven hot topics the one that interested them the most and wrote a few sentences on why they chose this hot topic. Participants also rated each hot topic on how important they believed it was. The hot topics were Immigration, Same Sex Marriage, Abortion, Climate Change, Legalization of Marijuana, Animal Rights, and Vaccinations.

After providing this information, participants completed some filler tasks (e.g., math problems, category sorting, etc.) to seemingly allow the collaboration portal time to create their profile and match them with a partner. Participants then “matched” with an ostensible partner. This partner was always described as “Sam M.,” but participants learned that no real names were being used to protect everyone’s privacy. Participants saw Sam’s supposed profile which featured a green leaf icon, indicated the platform they were using, their animal preference, and their short bio. All participants learned that Sam believed that climate change was an important topic and they volunteered at a local organization focused on sustainability. Participants also learned that Sam was either participating on the same platform as them or the other platform (Partner Platform Membership Condition), and that Sam either preferred the same animal as them or a different animal (Partner Animal Preference Condition). In addition, participants learned how Sam compared to others through a graphic that showed that there were equal numbers of participants from both platforms and animal preferences. However, they saw that Sam’s beliefs about climate change were much more important to Sam than to others in the system. This was done so participants knew how their partner’s attitude compared to the larger social group’s attitude.

After viewing this information, participants were led to believe that we wanted to provide time in between learning about their partner and their collaboration, so they answered some questions regarding the portal and more detailed beliefs on a few hot topics. Participants were assured that their feedback on the portal and their beliefs on the hot topics would *not* be shared with their partner. Participants completed the 15-item Climate Change Attitude Scale (Christensen and Knezek, 2015) and 10-items on the legalization of marijuana. The Climate Change Scale was our measure of social tuning, as those engaging in social tuning should endorse similar attitudes towards climate change as their partner (i.e., that climate change needs attention). Participants then completed a final questionnaire that assessed their memory for partner-relevant information, demographic information including gender and ethnicity, and any suspicions about the study. After completing these measures, participants learned there would be no collaboration with a partner. They were thanked for their participation, debriefed, and awarded monetary compensation (MTurk) or course credit (college).

## Results and discussion

To examine the effects of group membership on social tuning, analyses used a 2 (Partner Platform Membership: ingroup vs. outgroup) x 2 (Partner Animal Preference Membership: ingroup vs. outgroup) ANOVA. Since all participants learned that their partner believed climate change was a very important topic, higher scores indicate more social tuning with the interaction partner rather than the larger social group. Participants’ ratings of the importance of climate change as a hot topic prior to learning their partner’s stance was unsurprisingly highly correlated with, and a significant predictor of, their beliefs on climate change (*r* = 0.733, *p* < 0.001; *F* (1, 150) = 173.79, *p* < 0.001, *R*^2^ = 0.54, *R*^2^_adjusted_ = 0.53, 95% CI [0.65, 0.79]). Therefore, we covaried out this self-rating for analyses.

**Figure 1:**
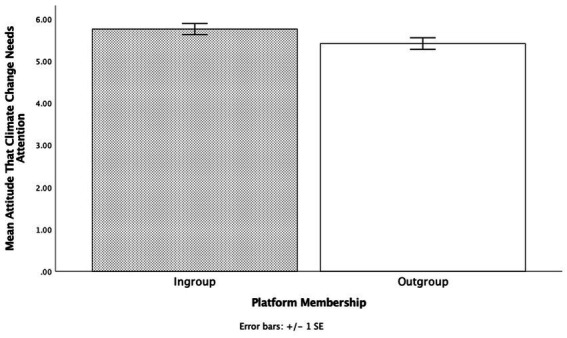
The effects of partner group membership through belonging to same or different group (MTurk or coolege) on beliefs that climate change needs attention in Experiment 1.

There was no main effect for Partner Animal Preference (*p* = 0.641) nor was there an interaction between the two partner group memberships (*p* = 0.700). However, there was a significant main effect for Partner Platform Membership, *F* (1, 147) = 5.88 *p* = 0.017, η_p_^2^ = 0.04, 95% CI [0.06, 0.59] (see Figure 1). When participants believed that their partner was from the same platform/ingroup member (*M* = 5.74, SD = 1.15) they reported that climate change needed more attention than when they believed their partner was from a different platform/outgroup member (*M* = 5.43, SD = 1.24). A bootstrap analysis with 1,000 samples replicated this main effect (*p* = 0.018).

Thus, the results show that individuals were more likely to engage in social tuning with their partner when they believed this partner was part of their ingroup compared to the outgroup. Moreover, the results suggest that sharing a membership in a group maybe more important than sharing a preference towards something. These results also indicate sharing multiple things in common does not necessarily increase the likelihood of engaging in social tuning.

## Experiment 2

Experiment 1 provides evidence that individuals who perceived similarity in a group membership with an interaction partner are more likely to engage in social tuning that those who are dissimilar with their partner. This occurred even though the interaction partner’s views differed from the larger social group. One limitation of Experiment 1 is that it held affiliative goals constant, and it is, therefore, unclear how affiliative motivation influences social tuning with an ingroup or outgroup partner whose views differ from the larger social group. Therefore, in Experiment 2, we directly test the role of affiliative motivation and perceived similarity (through group membership) on social tuning when the partner’s views differ from the larger social group.

## Method

### Participants

One hundred and fifteen individuals (69 males; 45 females; 1 unreported) from a private institution in the northeast United States participated for course credit and a chance to win a raffle prize. Sixty-two percent of the sample was White (71 White; 5 Black; 10 East Asian; 9 South Asian; 6 Multiracial; 13 Other; 1 unreported). Participants were from all undergraduate years (20% First Year, 27% Second Year, 25% Third Year, 27% Fourth Year, 1% Unreported). All participants gave informed consent. One participant did not complete the study and was removed from analyses. The analyses are based on 114 participants (68 males; 71 White).

### Design and materials

This experiment utilized a 2 (Affiliative Motivation: high or low) x 2 (Partner Group Membership: ingroup or outgroup) between-participants design.

#### Affiliative motivation manipulation

Adapting from past research (Sinclair et al., 2005a), we manipulated affiliative motivation through the length of time the participants believed they would interact with an ostensible partner: 5 min (low affiliative motivation) or 30 min (high affiliative motivation).

#### Partner group membership manipulation

We used the participants’ membership in Greek life to determine whether the ostensible interaction partner was part of their ingroup or outgroup. Prior to participating in any experiments, participants completed a pre-screening that included a question about whether they belonged to any Greek life organizations. If randomly assigned to the ingroup condition, then the ostensible partner was similar to the participant in Greek life affiliation (e.g., if the participant belonged to Greek life, the ostensible partner belonged to Greek life). If randomly assigned to the outgroup condition, then their ostensible partner was different than them in Greek life affiliation (e.g., if the participant belonged to Greek life, then the ostensible partner did not belong to Greek life).

To make the participants aware of the partner’s Greek life status, participants first wrote a short (few sentences) self-description for their partner to read regarding aspects about themselves such as group memberships, major, and activities. After completing their self-description, participants believed the computer was sending their description to their partner and that they would see their partner’s self-description. Participants then saw a partner description that read either: “I am a member of Greek life. I am still figuring out my major. I enjoy hanging out with friends.” or “I am a not a member of any Greek life organizations. I am still figuring out my major. I enjoy hanging out with friends.”

At the end of the study, participants also indicated whether they belonged to Greek life. We cross-checked this information with the pre-screening information as it was possible that a participant’s Greek Life membership could have changed from pre-screening to participating (e.g., joined a Greek Life organization). If there was a discrepancy, the group membership condition relied on the information the participant provided at the end of the study. Overall, 30% of the participants belonged to Greek life and 70% did not belong to Greek life. This is generally reflective of the student body at this institution as 37% belong to Greek life.

#### Perceived views of the partner and larger social group

Participants were led to believe that their partner held negative views towards drinking alcohol and this view was not held by other students at the same institution. To create this, participants viewed a list of scales and were told that the computer would randomly select a scale for them to complete and a scale for their partner to complete. The computer always asked participants to complete the Need for Closure Scale (Webster and Kruglanski, 1994), and it always led the participant to believe that the partner had been randomly selected to complete an Attitudes Towards Drinking Scale. After completing the Need for Closure Scale, participants thought their results on the Need for Closure scale were being sent to their partner and their partners results for the Attitudes Towards Drinking Scale were being sent to them. After a few minutes of the computer pretending to calculate the scores, participants learned that their ostensible partner’s score indicated that they had *less* favorable attitudes towards drinking than others who had previously taken the scale at their institution.

#### Social tuning measure

We measured participants’ self-reported drinking attitudes and behaviors to assess social tuning. We used 20 items from the College Drinking Attitudes Scale that utilized a 5-point Likert-Type scale (1 = very unlikely; 5 = very likely; Gonzalez, 1990). Participants indicated how likely they were to engage in different drinking-related activities, such as: “Always use alcohol as an addition to an activity rather than as the primary focus of attention”; “Set limits on how many drinks you are going to have on a night out or at a party”; “Drink alcohol to primarily get drunk.” Fifteen items were reverse scored to make higher numbers indicate *less* responsible drinking behaviors. The items were then averaged together, and the scores were standardized.

We also examined self-reported drinking behaviors. Items were adapted from the Student Alcohol Questionnaire (Engs, 1977). In this scale, participants indicated how often, on average, they drank beer, wine, or liquor on a 5-point Likert-Type scale (1 = “Every Day”; 2 = “Once a week” 3 = “Once a month”; 4 = “Every few months”; 5 = “Once a year”). Participants also reported the quantity of beer, wine, and liquor that they consumed in one setting on a 5-point Likert-Type scale (1 = “More than 6”; 2 = “5–6”; 3 = “3–4”; 4 = “1–2”; 5 = “less than 1”). All six items were reverse scored such that higher numbers meant more frequent drinking and more items consumed. The items were averaged together and standardized.

We created an Overall Drinking Attitudes and Behavior measure by averaging the standardized Attitudes Towards Drinking Scale, the Frequency of drinking beer, wine, and liquor, and the Quantity of beer, wine, and liquor consumed. Higher positive numbers indicate *less* responsible drinking attitudes and behavior (see Appendix B for all items and reverse scoring).

#### Follow-up and demographics

Participants also completed a questionnaire that they believed was a pre-interaction questionnaire. We assessed their memory for the scale their ostensible partner completed and their score, inquired into any suspicions they had during the study, and collected basic demographic information such as gender, ethnicity, year in school, and current Greek life status.

### Procedure

Participants believed they were participating in a study investigating what happened when people interacted with someone after hearing random pieces of information about them. Participants learned that they would first complete some tasks on the computer without their partner and then later in the study they would interact with their partner. Half the participants learned verbally that they would interact for 5 min (low affiliative motivation condition), and half the participants learned they would interact for 30 min (high affiliative motivation condition). This information was reiterated on the computer screen.

In the first task, participants briefly, in a few sentences, described themselves for their partner, including factors such as any group memberships (e.g., Greek life, clubs), major, hobbies, personality traits, etc. The computer program pretended to send their description to their ostensible partner and generate their partner’s self-description for them to review. Participants were randomly selected to view one of two possible self-descriptions: “I am a member of Greek life. I am still figuring out my major. I enjoy hanging out with friends” or “I am not a member of any Greek life organization. I am still figuring out my major. I enjoy hanging out with friends.” Hence, participants either learned their partner was part of their ingroup (e.g., they both belonged to Greek life or did not) or their outgroup (e.g., one belonged to Greek life and the other did not).

After reading their partner’s self-description, the experimenter informed participants that the computer would randomly select a questionnaire for them to complete, and their partner would also complete a randomly selected questionnaire. The experimenter also told participants that after completing their scale, the computer would generate and display their partner’s score on the scale they completed. Participants then saw a list of all the possible scales, but the computer always “randomly” selected the Need for Closure Scale (Webster and Kruglanski, 1994) for the participant. After completing the Need for Closure Scale, the computer displayed the list of scales again with the Attitudes towards Drinking Scale highlighted to indicate it was completed by the partner. The computer then generated the partner’s score. Participants always learned that their partner held *less* favorable attitudes towards drinking than the other participants in the same institution who had taken the same scale in our experiment. After learning their partner’s score in relation to the larger population, participants learned that their responses on any remaining scales would *not* be shared with their partner. Participants then answered questions about their attitudes towards drinking and frequency of drinking behavior, i.e., College Drinking Attitude Scale by Gonzalez (1990) and Student Alcohol Questionnaire by Engs (1977). This was our measure of social tuning. Participants also indicated their memory for partner-relevant information, any suspicions they had about the study, and demographic information including gender, ethnicity, year in school, and Greek life status. After completing these measures, participants learned there would be no interaction with a partner. They were thanked, debriefed, awarded course credit, and entered into a raffle.

## Results and discussion

To examine the effects of affiliative motivation and group membership on social tuning, analyses used a 2 (Affiliative Motivation: high vs. low) x 2 (Partner Group Membership: ingroup vs. outgroup) ANOVA. Since all participants learned that their partner held less favorable attitudes towards drinking, less frequent drinking behaviors indicates more social tuning with the interaction partner. All participants correctly remembered the scale their ostensible partner completed as well as their score on this measure. Participant gender had no effect on drinking attitudes and behaviors (*p* > 0.7).

**Figure 2:**
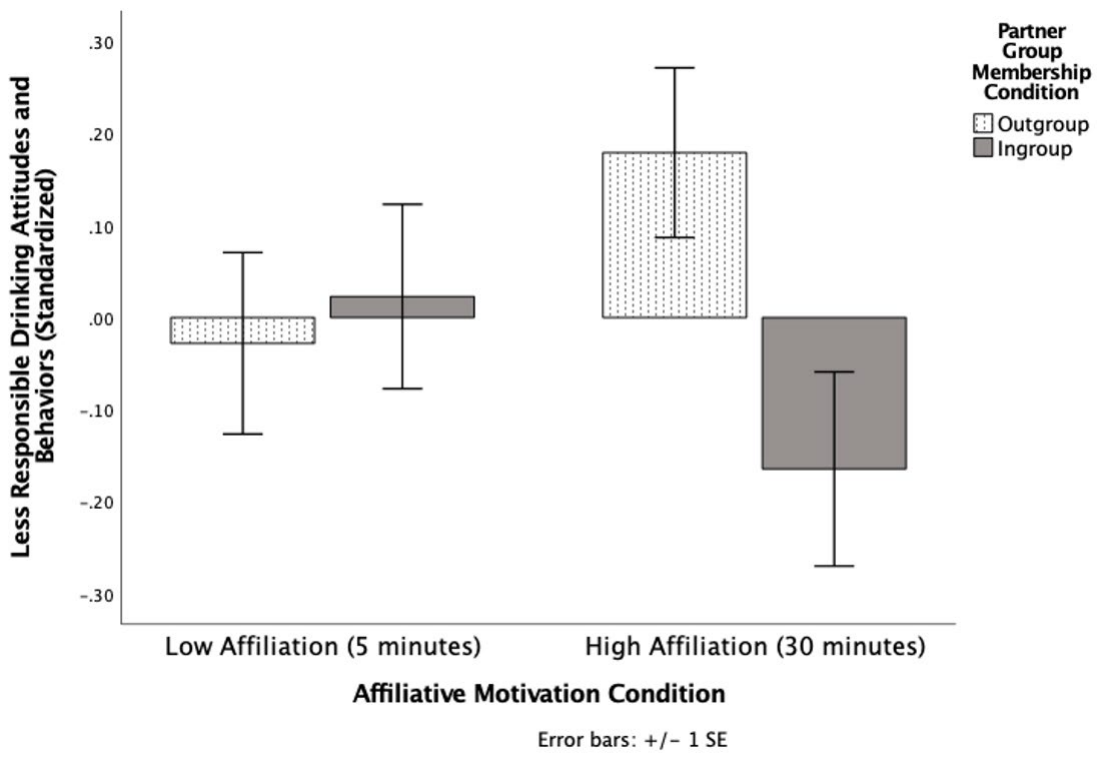
The effects of affiliative motivation and partner group membership on self-reported drinking attitudes and behavior (standardized) in Experiment 2.

There were no main effects for affiliative motivation (*p* = 0.922) or group membership (*p* = 0.146). However, there was a significant interaction between affiliative motivation and group membership, *F* (1, 110) = 3.90 *p* = 0.051, η_p_^2^ = 0.03 (see Figure 2).

Simple effects analyses showed that when participants had high affiliative motivation (interacting for 30 min), those who learned their partner was part of their ingroup (*M* = −0.17, SD = 0.59) engaged in social tuning by reporting more responsible drinking attitudes and behaviors (i.e., drinking less) than those who learned their partner was part of their outgroup (*M* = 0.18, SD = 0.50), *F* (1, 110) = 6.25, *p* = 0.014, η_p_^2^ = 0.05, 95% CI [0.07, 0.62]. This pattern held when a bootstrap analysis with 1,000 samples was applied (*p* = 0.020). However, participants with low affiliative motivation (i.e., interacting for 5 min) did not engage in social tuning regardless of whether their partner was in the ingroup (*M* = 0.02, SD = 0.53) or outgroup (*M* = −0.03, SD = 0.50), *p* = 0.727, η_p_^2^ = 0.00, 95% CI [−0.24, 0.34]. This pattern held bootstrapped with 1,000 samples (*p* = 0.710).

When the partner was part of the ingroup, affiliative motivation did not influence social tuning (*p* = 0.180, η_p_^2^ = 0.02, 95% CI [−0.09, 0.46]). This pattern held when a bootstrap analysis of 1,000 samples was applied (*p* = 0.208). When the partner was part of the outgroup, affiliative motivation did not influence social tuning (*p* = 0.152; η_p_^2^ = 0.02, 95% CI [−0.08, 0.49]). This pattern held when a bootstrap analysis with 1,000 samples was applied (*p* = 0.120).

An exploratory look at the means indicates that if these analyses would have been significant, interacting with an ingroup member with high affiliative motivation (*M* = −0.17 SD = 0.59) would have been more likely to endorse more responsible drinking than interacting with an ingroup member with low affiliative motivation (*M* = 0.02, SD = 0.53). However, the mean pattern looks different for outgroup partners, and, if anything, suggests potential anti-tuning. Interacting with an outgroup member with high affiliative motivation (*M* = 0.18, SD = 0.50) would have been less likely to endorse responsible drinking than interacting with an outgroup member with low affiliative motivation (*M* = −0.02, SD = 0.50). Overall, the results indicate that when participants had high affiliative motivation, they were more likely to engage in social tuning with their partner when the partner was part of their ingroup compared to the outgroup.

## Experiment 3

The results, thus far, show that an interaction partner’s views are more influential on an individual’s own beliefs than the larger social group, but only when the individual has the desire to get along with that partner and the partner is part of their ingroup. In Experiment 3, we seek to extend these studies by examining if these findings extend to implicit attitudes. Attitudes towards overweight individuals was chosen because the stigma towards overweight individuals is pervasive among men and women and even health professionals (Crandall, 1994; Teachman and Brownell, 2001; Wang et al., 2004; Brown, 2006; Brochu and Morrison, 2007). Furthermore, research consistently finds that overweight individuals do not exhibit any ingroup favorability towards other overweight individuals; therefore, participant’s own weight should not play a role in their expression of explicit or implicit attitudes (Crandall, 1994; Teachman and Brownell, 2001).

## Method

### Participants

A total of 69 individuals (24 females and 45 males) from a private institution in the northeastern United States participated and received course credit their participation. Seventy-eight percent of the sample was White (54 White; 1 Black; 4 East Asian; 3 South Asian; 3 Hispanic/Latino; 3 Multiracial; 1 Other). Participants were predominantly first- or second-year undergraduates (33% First Year, 33% Second Year, 19% Third Year, 15% Fourth Year). Three participants reported believing that their partner expressed favorable attitudes towards overweight individuals. Since they had incorrect perceived views, their data was removed from the analysis. Thus, the results are based off 66 participants. All participants gave informed consent.

### Design and materials

As in Experiment 2, this experiment utilized a 2 (Affiliative Motivation: high or low) x 2 (Partner Group Membership: ingroup or outgroup) between-participants design. In Experiment 3, we measured implicit and explicit attitudes towards the overweight.

#### Affiliative motivation manipulation

We used the same affiliative motivation manipulation as in Experiment 2 (length of interaction time).

#### Partner group membership manipulation

Participants learned their partner was part of their ingroup by being a student at the same school or their outgroup by being a student a different school in the same town.

#### Perceived views of the partner and larger social group

As in Experiments 1 and 2, participants always learned about their ostensible partner’s attitudes and how this compared to the larger social group. The ostensible partner’s score indicated that they held more negative or unfavorable attitudes towards overweight individuals than the other students at their school.

#### Social tuning measures

##### Explicit attitudes

We measured participant’s explicit views towards overweight people using Crandall’s (1994) Anti-fat Attitudes Scale. This scale consists of 10 questions that measure overall attitudes towards overweight individuals and includes questions such as: “I do not have many friends that are fat,” “Fat people tend to be fat pretty much through their own fault,” and “I worry about becoming fat.” The responses were measured on a 5-point Likert-type scale (1 = strongly disagree; 5 = strongly agree). Higher positive numbers indicate more negative attitudes towards overweight individuals (see Appendix C for all items).

##### Implicit attitudes

We measured implicit attitudes using the Overweight Implicit Association Test (IAT; Greenwald et al., 1998). In this IAT, participants focused on the center of the screen and categorized words as being “pleasant” or “unpleasant” (e.g., “happy” or “rotten”) and pictures as being “normal” or “overweight” as quickly as possible (all materials used in this IAT were from Nosek et al., 2007). Participants first categorized one attribute-pair (e.g., pleasant/unpleasant or overweight/normal). Then, they completed trials for the second attribute-pair. After categorizing each attribute-pair individually, participants completed trials where they categorize both attribute-pairs at the same time (e.g., pleasant/normal; unpleasant/overweight). The reaction times of the categorizations were used to compute the strength of the association between the different pairings (see Greenwald et al., 2003). The category positions were counterbalanced across participants. Higher negative scores indicate more negative/unpleasant associations towards overweight individuals.

#### Follow-up and demographics

As in Experiments 1 and 2, we assessed memory for the scale the partner completed and score, inquired into any suspicions, and collected basic demographic information such as gender, ethnicity, and year in school.

### Procedure

The procedure was very similar to Experiment 2. Participants learned the study investigated social interactions that occur when individuals have different information about each other and that they would complete several initial tasks and then work with a partner. Half the participants were randomly selected to learn that that their partner was from the same school (ingroup), and the other half learned their partner was from a different, though local, school (outgroup). This served as the partner group membership manipulation. As in Experiment 2, participants believed they would be working with their partner for either 5 min (low affiliative motivation) or 30 min (high affiliative motivation). The computer “randomly” assigned all participants to complete the Need for Closure Scale (Webster and Kruglanski, 1994). Participants believed that the ostensible partner completed a Body Attitudes scale, and that their partner’s score indicated that they had more unfavorable towards overweight individuals than others from their school. After learning this information, participants completed the Overweight Implicit Associations Test (IAT, Greenwald et al., 1998), Crandall’s (1994) Anti-fat Attitudes Scale, and a final questionnaire that assessed memory for partner-relevant information, demographic information (e.g., gender, ethnicity), and any suspicions. Participants learned there would be no interaction, were thanked, debriefed, and awarded credit.

## Results and discussion

To examine the effects of affiliative motivation and group membership on social tuning, analyses used a 2 (Affiliative Motivation: high vs. low) x 2 (Group Membership: ingroup vs. outgroup) ANOVA. Since all participants learned that their partner held more unfavorable views of overweight individuals than other students at their school, more unfavorable attitudes indicate more social tuning with the interaction partner. Participant gender did not influence the results, *p*s > 0.1.

### Explicit attitudes

**Figure 3:**
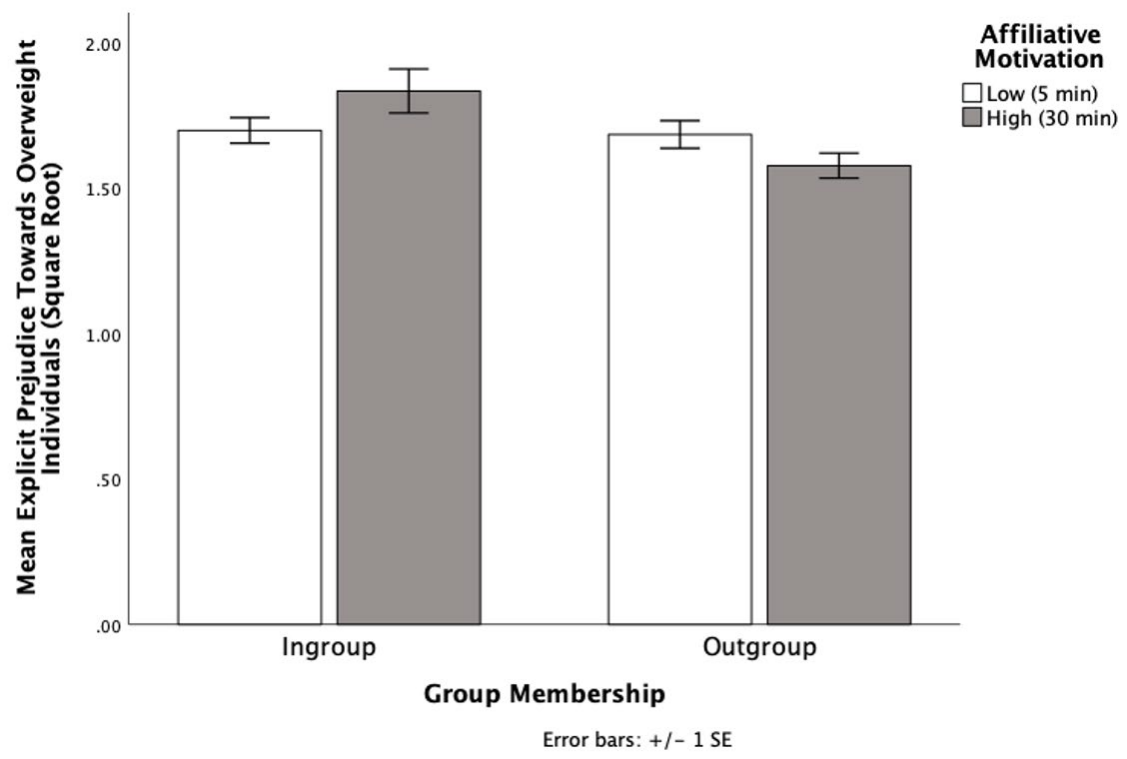
The effects of group membership and affiliative motivation on explicit attitudes towards overweight individuals in Experiment 3.

Descriptive analyses showed a moderate positive skew in explicit measure. We applied a square root transformation to adjust for this skew (Howell, 2007). Higher positive numbers indicate more stereotypic attitudes towards overweight individuals. There was no main effect found for affiliative motivation, *p* = 0.785, η_p_^2^ = 0.00. However, there was a main effect for group membership *F* (1, 62) = 6.81 *p* = 0.011, η_p_^2^ = 0.10, 95% CI [0.03, 0.24]. Those paired with ingroup partners (*M* = 1.76, SD *= *0.23) tuned more towards their partners prejudiced attitudes than those paired with outgroup partners (*M =* 1.63, SD *= *0.20). This main effect is qualified by a significant interaction between affiliative motivation and group membership, *F* (1, 62) = 5.48, *p* = 0.022, η_p_^2^ = 0.08 (see Figure 3).

Simple effects analyses showed that when participants had high affiliative motivation, they were more likely to tune towards the prejudiced attitudes of their partner when the partner was part of their ingroup (*M* = 1.84, SD = 0.27) as opposed to part of their outgroup (*M* = 1.58, SD = 0.18), *F* (1, 62) = 11.46, *p* = 0.001, η_p_^2^ = 0.16, 95% CI [0.11, 0.41]. The pattern held when a bootstrap analysis with 1,000 samples was applied (*p* = 0.006). For ingroup partners, participants with high affiliative motivation (*M* = 1.84, SD = 0.27) marginally tuned towards the prejudiced attitudes of their interaction partner than those with low affiliative motivation (*M* = 1.70, SD = 0.18), *F* (1, 62) = 3.04, *p* = 0.086, η_p_^2^ = 0.05, 95% CI [−0.02, 0.29]. This remained marginal/not significant bootstrapped with 1,000 samples (*p* = 0.142).

However, when participants had low affiliative motivation, there was no difference in explicit attitudes when their partner was from their ingroup (*M* = 1.70, SD = 0.18) than the outgroup (*M* = 1.69; SD = 0.21), *p* = 0.845, 95% CI [−0.13, 0.16]. The pattern held when a bootstrap analysis with 1,000 samples was applied (*p* = 0.831). Also, when the partner was an outgroup member, there was no difference in explicit attitudes when the participant had high affiliative motivation (*M* = 1.58; SD = 0.18) compared to low affiliative motivation (*M* = 1.69; SD = 0.21), *p* = 0.123, 95% CI [−0.03, 0.24]. This pattern held when bootstrapped with 1,000 samples (*p* = 0.095).

### Implicit attitudes

**Figure 4:**
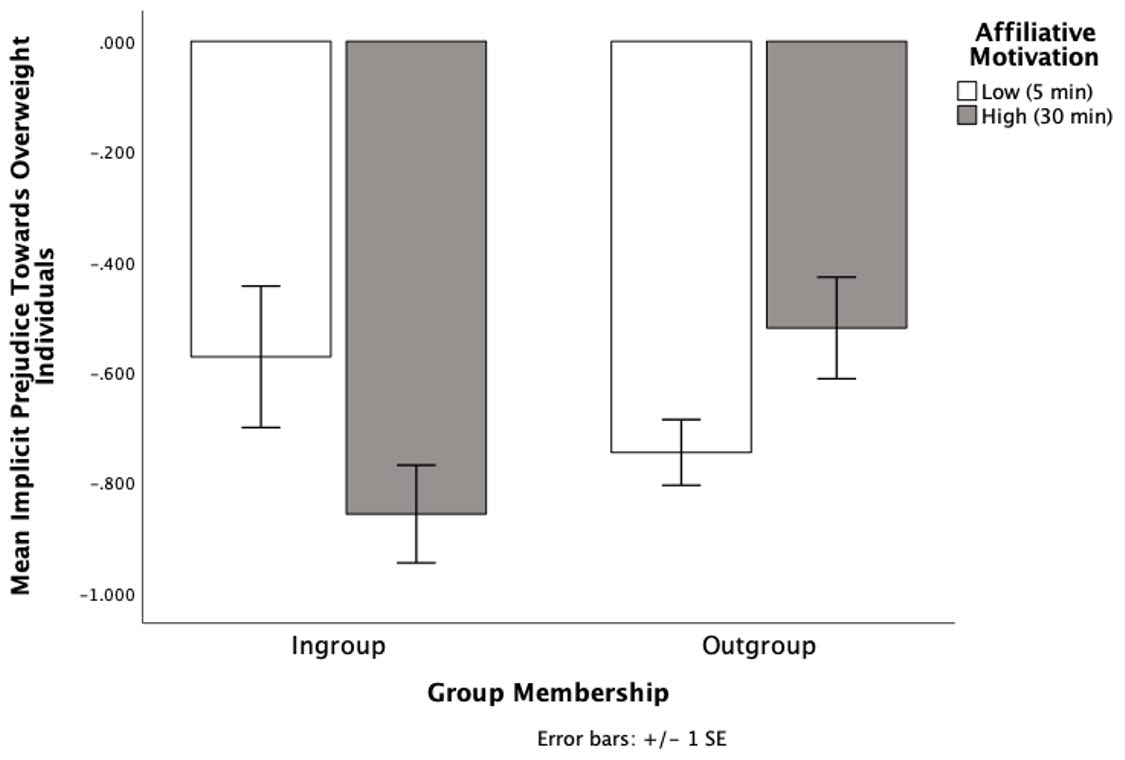
The effects of group membership and affiliative motivation on implicit attitudes towards overweight individuals in Experiment 3.

Looking at implicit attitudes, higher negative numbers (i.e., −1, −2) indicate more stereotypic attitudes towards overweight individuals and higher positive numbers indicate more egalitarian attitudes towards overweight individuals. There were no main effects for affiliative motivation (*p* = 0.763) or group membership (*p* = 0.407). But there was a significant interaction between affiliative motivation and group membership on implicit attitudes, *F* (1, 61) = 6.78, *p* = 0.012, η_p_^2^ = 0.10 (see Figure 4).

Simple effects analyses showed that of the participants with high affiliative motivation, those who learned their partner was part of their ingroup (*M* = −0.86, SD = 0.32) tuned more towards the prejudiced attitudes of their interaction partner than those who learned their partner was part of the outgroup (*M* = −0.52; SD = 0.37), *F* (1, 61) = 5.32, *p* = 0.025, η_p_^2^ = 0.08, 95% CI [0.05, 0.63]. The pattern held when a bootstrap analysis with 1,000 samples was applied (*p* = 0.010). When participants learned their partner was a member of their ingroup, those with high affiliative motivation (*M* = −0.86, SD = 0.32) tuned more towards the prejudiced attitudes of their interaction partner than those with low affiliative motivation (*M* = −0.57, SD = 0.54), *F* (1, 61) = 3.99, *p* = 0.050, η_p_^2^ = 0.06, 95% CI [0.00, 0.57]. This effect became marginal when a bootstrap analysis with 1,000 samples was applied (*p* = 0.071).

However, when participants had low affiliative motivation, there was no difference in implicit attitudes when their partner was from their ingroup (*M* = −0.57; SD = 0.54) than the outgroup (*M* = −0.75; SD = 0.25), *p* = 0.189, 95% CI [−0.09, 0.44]. This held after a bootstrap analysis with 1,000 samples (*p* = 0.210). Also, when the partner was an outgroup member, there was no difference in implicit attitudes when the participant had high affiliative motivation (*M* = −0.51; SD = 0.37) compared to low affiliative motivation (*M* = −0.75; SD = 0.25), *p* = 0.099, 95% CI [−0.04, 0.50]. This effect became marginal when a bootstrap analysis with 1,000 samples was applied (*p* = 0.056), indicating a potential anti-tuning effect where when interacting with an outgroup member, participants expressed *less* implicit prejudice when they had high affiliative motivation (*M* = −0.52, SD = 0.37) compared to low affiliative motivation (*M* = −0.75; SD = 0.25).

## Experiment 4

Experiment 4 uses the same methodology as Experiment 3 but investigates whether affiliative motivation and group membership influence social tuning when the ostensible partner endorses more positive attitudes towards weight than the larger social group.

## Method

### Participants

A total of 93 individuals (50 females, 40 males, 1 Other, and 1 who did not disclose) from a private institution in the northeastern United States participated and received course credit their participation. Sixty-five percent of the participants were White (24% Asian/South Asian, 4% Latinx, 3% Black, 3% multi-racial, 1 did not report). Participants were predominantly first- or second-year undergraduates (35% First Year, 28% Second Year, 15% Third Year, 20% Fourth Year, 1% Graduate Student, 1% Not in School). All participants gave informed consent.

### Design and Procedure

Experiment 4 used the same methods as Experiment 3. The only difference was the perceived views of the ostensible partner which were more positive or favorable towards overweight than other individuals at their school. Thus, participants were randomly assigned to learn that their ostensible partner was from the same school (ingroup) or a different local school (outgroup). Participants believed they would be working with this partner for 5 min (low affiliative motivation) or 30 min (high affiliative motivation). Participants were then “randomly” assigned to complete the Need for Closure Scale (Webster and Kruglanski, 1994), and believed their ostensible partner completed a Body Attitudes scale. However, in Experiment 4, the partner’s score indicated that they had more favorable towards overweight individuals than others from their school. Participants then completed the Overweight Implicit Associations Test (IAT, Greenwald et al., 1998), Crandall’s (1994) Anti-fat Attitudes Scale, and a final questionnaire that assessed memory for partner-relevant information, demographic information (e.g., gender, ethnicity), and any suspicions. Participants learned there would be no interaction, were thanked, debriefed, and awarded credit.

## Results and discussion

As in Experiment 3, analyses used a 2 (Affiliative Motivation: high vs. low) x 2 (Group Membership: ingroup vs. outgroup) ANOVA. Since all participants learned that their partner held more favorable views of overweight individuals than other students at their school, more favorable attitudes indicate more social tuning with the interaction partner. Participant gender influenced the results (*p* > 0.01); therefore, it was a covariate in the analyses.

### Explicit attitudes

Descriptive analyses revealed that the Fear subscale on the Anti-Fat Attitudes Scale (Crandall, 1994) behaved differently than the Dislike and Willpower subscales. The Fear subscale has three items about fears relating to the individual gaining weight [e.g., “I worry about becoming fat”). The Dislike and Willpower subscales are perceptions of overweight individuals (e.g., “Fat people tend to be fat pretty much through their own fault (willpower)” or “Fat people make me feel somewhat uncomfortable (dislike)].” Therefore, we conducted two analyses: one for the group-based beliefs (Dislike and Willpower subscales) and one for self-based beliefs (i.e., Fear subscales).

#### Group-based explicit attitudes

**Figure 5:**
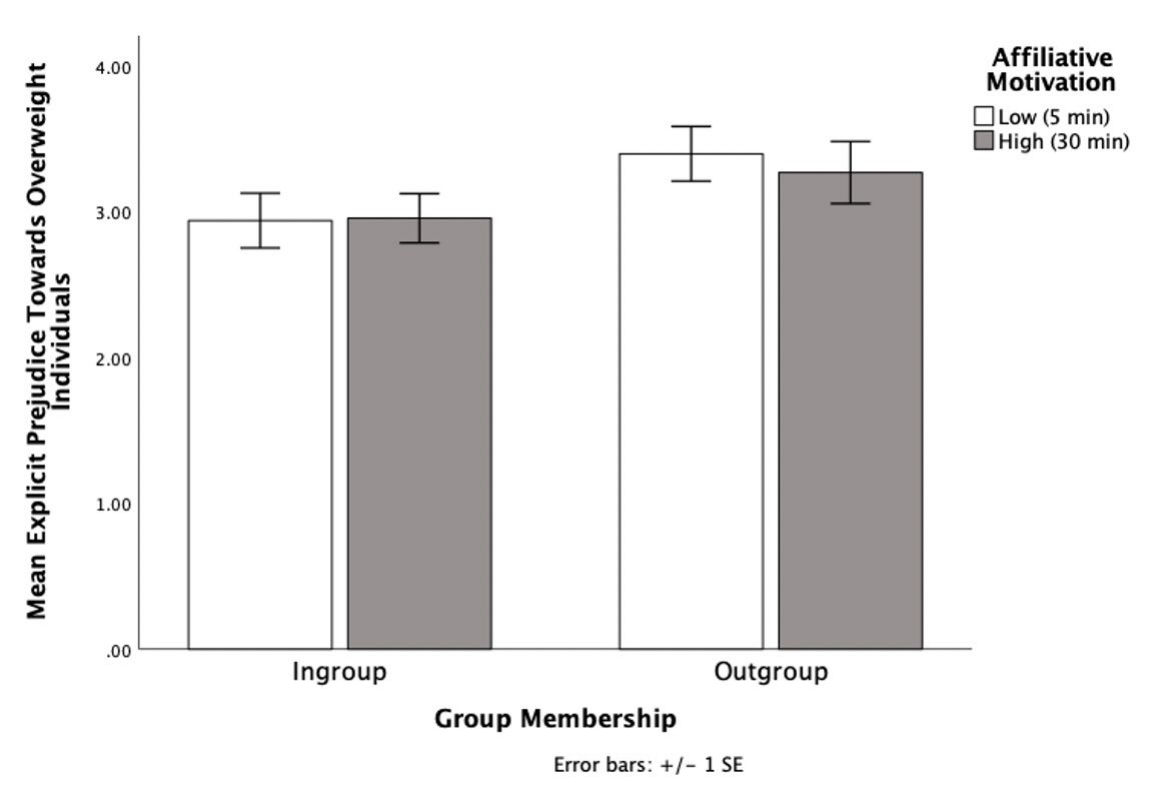
The effects of group membership and affiliative motivation on explicit attitudes towards overweight individuals in Experiment 4.

While there was no main effect for affiliative motivation (*p* = 0.675, η_p_^2^ = 0.00), there was a main effect for group membership *F* (1, 84) = 3.99 *p* = 0.049, η_p_^2^ = 0.05, 95% CI [0.00, 0.72]. Those paired with ingroup partners (*M* = 2.95, SD *= *0.87) tuned more towards their partners egalitarian attitudes towards overweight individuals than those paired with outgroup partners (*M =* 3.34, SD *= *0.89). This main effect held when a bootstrap analysis with 1,000 samples was applied (*p* = 0.051). However, unlike Experiment 3, there was no significant interaction between affiliative motivation and group membership, *p* = 0.578, η_p_^2^ = 0.00. See Figure 5.

#### Individual-related explicit attitudes

As for individual-based attitudes, there was no main effect found for affiliative motivation, *p* = 0.120, η_p_^2^ = 0.03. However, there was a main effect for group membership *F* (1, 84) = 3.86, *p* = 0.053, η_p_^2^ = 0.05, 95% CI [−0.01, 1.4]. Those paired with ingroup partners (*M* = 4.48, SD *=* 1.62) expressed more fears about becoming overweight compared to those paired with outgroup partners (*M =* 3.74, SD *=* 1.70). This main effect became marginal when a bootstrap analysis with 1,000 samples was applied (*p* = 0.065). There was no significant interaction between affiliative motivation and group membership, *p* = 0.457, η_p_^2^ = 0.01.

### Implicit attitudes

**Figure 6:**
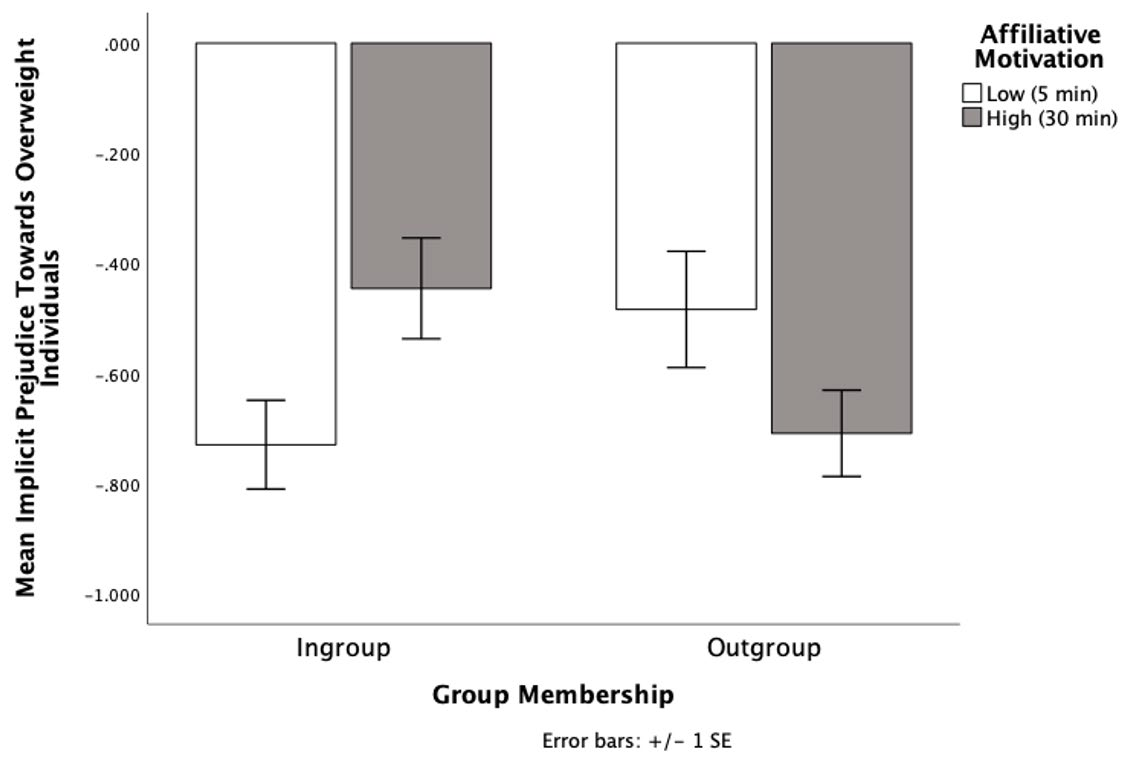
The effects of group membership and affiliative motivation on implicit attitudes towards overweight individuals in Experiment 4.

Looking at implicit attitudes, higher positive numbers (i.e., 1, 2) indicate more egalitarian attitudes towards overweight individuals. Analyses revealed four outliers on the IAT which were removed for the analysis. There were no main effects for affiliative motivation (*p* = 0.663) or group membership (*p* = 0.990). But there was a significant interaction between affiliative motivation and group membership on implicit attitudes, *F* (1, 80) = 7.58, *p* = 0.007, η_p_^2^ = 0.09 (see Figure 6).

Simple effects analyses showed that of the participants with high affiliative motivation, those who learned their partner was part of their ingroup (*M* = −0.45, *SD* = 0.47) tuned more towards the egalitarian attitudes of their interaction partner than those who learned their partner was part of the outgroup (*M* = −0.71; SD = 0.33), *F* (1, 80) = 3.82, *p* = 0.05, η_p_^2^ = 0.054, 95% CI [−0.01, 0.50]. The pattern held when a bootstrap analysis with 1,000 samples was applied (*p* = 0.049). When participants learned their partner was a member of their ingroup, those with high affiliative motivation (*M* = −0.45, SD = 0.47) tuned more towards the egalitarian attitudes of their interaction partner than those with low affiliative motivation (*M* = −0.72, SD = 0.40), *F* (1, 80) = 5.67, *p* = 0.020, η_p_^2^ = 0.07, 95% CI [0.05, 0.53]. This effect remained when a bootstrapped with 1,000 samples (*p* = 0.016).

However, when participants had low affiliative motivation, those with an ingroup partner (*M* = −0.73; SD = 0.37) were marginally more likely to anti-tune than those with an outgroup partner (*M* = −0.48; SD = 0.47), *F* (1, 80) = 3.76, *p* = 0.056, η_p_^2^ = 0.05, 95% CI [−0.01, 0.51]. This held after a bootstrap analysis with 1,000 samples (*p* = 0.053). However, when the partner was an outgroup member, there was no difference in implicit attitudes when the participant had high affiliative motivation (*M* = −0.71; SD = 0.33) compared to low affiliative motivation (*M* = −0.48; SD = 0.47), *p* = 0.123, 95% CI [−0.06, 0.48]. This non-significant effect remained after being bootstrapped with 1,000 samples (*p* = 0.104).

## General discussion

Across four experiments, the results consistently demonstrated that social tuning with an interaction partner will occur even when the interaction partner’s beliefs differ greatly from the larger social group. More specifically, the results demonstrated that individuals with high affiliative motivation were more likely to divert away from social consensus and engage in social tuning with their ingroup than outgroup partner. This occurred for explicit attitudes (Experiments 1–3) and implicit attitudes (Experiments 3 and 4).

These findings are consistent with the tenets of both Social Learning Theory and Shared Reality Theory as they reiterate that identifying or sharing something in common with another person (e.g., a social model) as well as having a motivation to get along with interaction partners are important factors predicting when individuals are likely to engage in social learning or experience shared reality (XXXBandura, 1969; Hardin and Conley, 2001). The findings are also consistent with the affiliative social tuning hypothesis (Sinclair et al., 2005a,b; Skorinko and Sinclair, 2018) because believing an interaction partner was similar in terms of group membership increased the likelihood of social tuning, especially when affiliative motivation was high.

One caveat to this finding was the explicit weight-based attitudes in Experiment 4 when the partner endorsed positive weight-based attitudes. In this instance, group membership, more so than affiliative motivation encouraged social tuning. More specifically, when interacting with an ingroup member, participants social tuned by expressing more favorable attitudes towards overweight individuals. This finding is similar to Experiment 1 when affiliative motivation was held constant. However, this is not as consistent with Experiments 2 and 3 where those with high affiliative motivation engaged in social tuning of explicit attitudes with an ingroup member more than an outgroup member. An exploratory look at the means shows a similar pattern for group based explicit attitudes Experiment 4; however, it was not significant. Yet, for implicit attitudes, participants in Experiments 3 and 4 engaged in social tuning based on affiliative motivation and group membership, as those who had high affiliative motivation tuned more towards an ingroup than outgroup member. Thus, overall, the pattern of results is similar.

In addition, the type of explicit attitude mattered in Experiment 4 as social tuning did not occur for attitudes related to participant’s own body image (e.g., if they gained weight). Rather, individuals interacting with an ingroup member expressed more fears about gaining weight than those interacting with an outgroup member. Yet, in Experiment 3, when the interaction partner endorsed negative weight-based attitudes, there was no differences based on the type of attitude (overweight as a group; self/individual). And, again, social tuning occurred for implicit attitudes as predicted between affiliative motivation and group membership in Experiments 3 and 4.

Experiments 3 and 4 used the same methodology except for the attitude endorsed by the partner towards overweight individuals (negative in Experiment 3 and positive in Experiment 4). However, Experiment 4 had a larger percentage of female participants than Experiment 3 and participant gender was a significant factor in Experiment 4, but not Experiments 1–3. Male participants in Experiment 4 expressed significantly more negative views towards overweight individuals than female participants, but female participants tended to express greater fears in becoming overweight. Therefore, the difference in explicit responses may be due to the pervasive, yet changing, nature of weight-based stigmas (Puhl and Heuer, 2009). Since weight-based stigma is still pervasive it may be expressed explicitly, but you may be more motivated to express such negative sentiments if you have a strong desire to get along with an interaction partner, especially one that is similar to you. However, when the partner expresses positive attitudes towards overweight individuals, it may not require the same level of affiliation to endorse positive/favorable views of others when interacting with someone.

The findings likely reflect the complicated relationship between gender and body image. Past work shows that when women are primed to think about their bodies (e.g., putting on a swimsuit compared to a sweater), they are more likely to engage in self-objectification than men (e.g., Fredrickson et al., 1998; Hebl et al., 2004). Likewise, women exposed to images of celebrities and thin peers on social media platforms are more likely to express more body dissatisfaction than those who saw neutral images (Brown and Tiggemann, 2016). Thus, in the current work, learning that one’s partner endorses positive body attitudes may encourage participants to endorse similar views when the interaction partner is similar. However, applying those positive body attitudes to oneself, especially for women, may be harder to do—especially when someone learns their partner is more positive than the general public. This social consensus information may have inadvertently served as a prime about negative societal body image attitudes and in returned acted like a swimsuit or viewing a thin celebrity and increased fears of gaining weight. Therefore, future research should continue to examine social tuning for weight-based stigma in relation to gender identity to further understand and unpack these differences in the findings.

The results from these four experiments contradict the findings from Sinclair et al. (2005b) where they found that group membership (based on experimenter’s race) had no influence on social tuning. We do not believe that either result is in error. Rather, we believe different situational mechanisms are at play. In the original study (Sinclair et al., 2005b), participants were run in groups rather than one-on-one interactions. We believe that this is an important distinction because group membership is likely to be much more salient and dominant when an interaction is dyadic in nature, especially in situations where affiliative motivation is high and social tuning is likely to occur. Future research should examine whether group size influences the effects of perceived similarity through group membership on social tuning.

In addition, the original study by Sinclair et al. (2005a) used the experimenter as the interaction partner. Past work consistently finds that participants who have high affiliative or epistemic motivation will tune towards an interaction partner that is an experimenter (Sinclair et al., 2005a,b; Lun et al., 2007; Skorinko and Sinclair, 2018). The findings from the current work suggest that perceived similarity through group membership may also be effective in eliciting social tuning when the interaction partner is a peer rather than in a position of perceived power (e.g., experimenter). Bandura (1969) argues that we are more likely to see those in higher social status or social power (e.g., celebrities, experts, etc.) as social models. Therefore, it is possible that the social status or social power that comes with being an experimenter plays a greater role than perceived similarity in a social interaction, but when interacting with someone who is on a more level playing field status/power wise than perceived similarity becomes a more important factor. Future research should explore how social hierarchies through social status and/or social power influence the likelihood that perceived similarity predicts social learning and social tuning.

In research like the current work, a concern raised is whether the results really represent the construct being measured (i.e., social tuning) or self-presentation. We contend that self-presentation is unlikely an explanation for the findings for two reasons. First, we took self-presentation into account as we designed our experiments. As such, we made it very clear to participants what information was shared with their partner and what information was not shared. Since participants knew that their responses to the attitude measures would *not* be shared with their partner, their motivation to self-present should be limited. Second, past research shows that social tuning is not due to strategic self-presentation (Sinclair et al., 2005a,b). We see, as in past research, social tuning occurs for implicit attitudes (Experiments 3 and 4) indicating that strategic self-presentation is unlikely to be occurring. It is also possible that the results found are, in part, due to the clarity in which the partner’s attitude and the larger social groups attitudes were expressed, as past work has found that clarity in social norms of expression of prejudice influenced individual beliefs (Zitek and Hebl, 2006).

Affiliative motivation is not the only motivation that encourages social tuning to occur. Research also finds that epistemic motivation (e.g., the desire to gain information; see Lun et al., 2007), perspective taking (e.g., putting yourself in the shoes of others; Skorinko et al., 2023), and cultural background/mindset (Skorinko et al., 2015) also predict when social tuning is likely to occur. From both a social learning and a shared reality standpoint, it seems like perceived similarity, especially through group membership, might also play a role when these different motivations are activated as well. For example, if an individual is experiencing epistemic motivation because they want to gain more information about how their partner perceives something or someone, then is also seems likely that the perceived similarity (or lack thereof) with this partner would influence their likelihood to social tune. Thus, future research should examine the role the perceived similarity, especially through group membership, plays when other motivations to engage in social tuning are active. This work should also investigate whether social consensus (Stangor et al., 2001) inhibits social tuning when these different motivations are active as well.

Finally, Bandura (1969) argued that perceived similarity was important to social learning but that it, in and of itself, may not be enough to create identification with a social model and some other factors (such as motivation) may be needed to for social learning to occur. This contention may relate to the difference between sharing a surface- or a deep-level characteristics with someone else (Harrison et al., 1998). Surface-level characteristics include observable cues such as gender, race, and age. Deep-level characteristics include non-observable cues such as attitudes, beliefs, skill sets, and values. While both surface- and deep-level similarity result in attraction toward individuals, surface-level similarities are a weaker predictor of positive evaluations and reducing bias than deep-level similarities (Swim, 1993; Ensher et al., 2002). For instance, one study found that attitudinal similarity was a better predictor of a mentor’s satisfaction and support than demographic similarity (Ensher et al., 2002). In the current work, participants learn not only about a surface-level characteristic about their partner (i.e., their group membership) but they also learn about deeper-level characteristics through the attitude they endorse. Other work argues that differences in group types influences the effect they have on a person (e.g., a minimal/less consequential group to a more consequential group; Blocker and McIntosh, 2017). Therefore, future research may want to disentangle the differences between surface and deep-level characteristics and different group types and investigate how these factors influence the social tuning process.

In summary, the current research extends the affiliative social tuning hypothesis by demonstrating that the effects of affiliative motivation on social tuning are amplified when the interaction partner belongs to the ingroup rather than the outgroup. Furthermore, this work provides evidence that the beliefs of an immediate social interaction partner can, at times, be more influential in an individual’s personal beliefs, than the larger social groups beliefs. This work aligns with Bandura’s Social Learning Theory because it shows that both identification through perceived similarity and the motivation to get along with someone influence whether an individual aligns their views with an interaction partner or the larger social group. These findings have larger implications for the transmission of attitudes, especially intergroup attitudes because these findings imply that the transmission of prejudiced or egalitarian attitudes are likely to be greater when an individual has a desire to interact with an ingroup member.

## Data availability statement

The raw data supporting the conclusions of this article will be made available by the authors, without undue reservation.

## Ethics statement

The studies involving human participants were reviewed and approved by Worcester Polytechnic Institute Institutional Review Board (IRB). The patients/participants provided their written informed consent to participate in this study.

## Author contributions

JS led the experimental design, analyses, and manuscript writing. M-SJ and AD assisted in the manuscript writing. The remaining authors all contributed equally to this work. NE, MF, JH, and GG helped design, run, and assisted in analyses for E1. SM, CM, and LR helped design, run, and assisted in the analyses for E2. KHeather helped run and conducted analyses for E2. SS helped design, run, and assisted in the analyses for E3. TJ, DV, AV, MK, MaS, KR, KHeyer, AI, MrS, and KHo helped run and assisted in the analyses for E3. CP and EB conducted E4 and assisted with manuscript writing. All authors contributed to the article and approved the submitted version.

## Conflict of interest

The authors declare that the research was conducted in the absence of any commercial or financial relationships that could be construed as a potential conflict of interest.

## Publisher’s note

All claims expressed in this article are solely those of the authors and do not necessarily represent those of their affiliated organizations, or those of the publisher, the editors and the reviewers. Any product that may be evaluated in this article, or claim that may be made by its manufacturer, is not guaranteed or endorsed by the publisher.
