# Supplementary material for: You and me versus the rest of the world: the effects of affiliative motivation and ingroup partner status on social tuning
Source: Front Psychol. 2023 Aug 17;14:1060166. doi: 10.3389/fpsyg.2023.1060166 (PMC10470833; doi:10.3389/fpsyg.2023.1060166)
Supplement: Supplementary file 1 [file Data_Sheet_1.docx]

**Appendix A**

**Items Adapted from The Climate Change Attitudes Scale (Christensen & Knezek, 2015)**

Utilizes a 7-point Likert-Type Scale (1 = Strongly Disagree; 7 = Strongly Agree)

1. I believe our climate is changing.
2. I am concerned about global climate change.
3. I believe there is evidence of global climate change.
4. Global climate change will impact our environment in the next 10 years.
5. Global climate change will impact future generations.
6. The actions of individuals can make a positive difference in global climate change.
7. Human activities cause global climate change.
8. Climate change has a negative effect on our lives.
9. We cannot do anything to stop global climate change. (RECODED)
10. I can do my part to make the world a better place for future generations.
11. Knowing about environmental problems and issues is important to me.
12. I think most concerns about environmental problems have been exaggerated. (RECODED)
13. Things I do have no effect on the quality of the environment. (RECODED)
14. It is a waste of time to work to solve environmental problems. (RECODED)
15. There is not much I can do that will help solve environmental problems. (RECODED)

**Appendix B**

**Items Adapted from the Gonzales (1990) College Drinking Attitudes Scale**

Utilizes a 5-point Likert-Type Scale (1 = Very Unlikely; 5 = Very Likely)

1. Always use alcohol as an addition to an activity rather than as the primary focus of attention. (RECODED)
2. Rationalize drinking by such comments as 'I just need one more to relax' or ‘How about one for the road'.
3. Provide non-alcoholic alternative drinks; fruit juices, unspiked punch, coffee, or tea at your party. (RECODED)
4. Set limits on how many drinks you`re going to have on a night out or at a party. (RECODED)
5. Gulp drinks for the stronger and faster effect.
6. Respect a person who chooses to abstain from alcohol. (RECODED)
7. Drink alone from a desire to escape boredom or loneliness.
8. Tell a friend that there is nothing funny about being drunk when he or she is bragging about drinking. (RECODED)
9. Seriously think about the problems of alcohol abuse. (RECODED)
10. Talk about how to use alcohol responsibly with your roommate or close friend. (RECODED)
11. Express displeasure to someone who has had too much to drink at your party. (RECODED)
12. Provide transportation or overnight accommodations to someone who is unable to drive safely after drinking at your party. (RECODED)
13. Always celebrate by drinking when things go well for you.
14. Provide food when you`re hosting a party or social event where alcohol is being served. (RECODED)
15. Discourage a date or friend who is under the influence of alcohol from driving. (RECODED)
16. Get involved in trying to help a friend or associate who has a drinking problem. (RECODED)
17. Drink alcohol primarily to get drunk.
18. Know and stay within your personal drinking limit based on body weight if you are going to drive. (RECODED)
19. Not be insistent about 'refreshing' or refilling drinks. (RECODED)
20. Seek help if you thought you had a drinking problem. (RECODED)

**Items Adapted From the Student Alcohol Questionnaire (Engs, 1977)**

*Note: All items were reverse scored so that higher numbers indicated drinking more often and consuming more alcoholic beverages.*

1. How often, on average, do you typically have a beer?
   1. 1="Every Day”, 2 =" Once a week", 3= "Once a month", 4= "Every few months", 5="Once a year or less"
2. When you drink beer, how much, on the average, do you usually drink at any one time (in terms of cans or tavern glasses)?
   1. 1="More than 6", 2 ="5-6", 3= "3-4”, 4= "1-2", 5="less than 1"
3. How often, on average, do you typically have wine?
   1. 1="Every Day”, 2 =" Once a week", 3= "Once a month", 4= "Every few months", 5="Once a year or less"
4. When you drink wine, how much, on the average, do you usually drink at any one time (in terms of standard wine glasses)?
   1. 1="More than 6", 2 ="5-6", 3= "3-4”, 4= "1-2", 5="less than 1"
5. Next we would like to ask you about liquors and spirits (whiskey, gin, vodka, mixed drinks, etc.). How often do you usually have a drink of liquor?
   1. 1="Every Day”, 2 =" Once a week", 3= "Once a month", 4= "Every few months", 5="Once a year or less"
6. When you drink liquor, how many drinks, on the average, do you usually drink at any one time
   1. 1="More than 6", 2 ="5-6", 3= "3-4”, 4= "1-2", 5="less than 1"

**Appendix C**

**Items Adapted from Crandall’s (1994) Anti-fat Attitudes Scale**

Utilizes a 5-point Likert-Type Scale (1 = Strongly Disagree; 5 = Strongly Agree)

1. People who weigh too much could lose at least some of their weight through a little exercise.
2. I worry about becoming fat.
3. Fat people tend to be fat pretty much through their own fault.
4. I tend to think that people who are overweight are a little untrustworthy.
5. Fat people make me feel somewhat uncomfortable.
6. I feel disgusted with myself when I gain weight.
7. One of the worst things that could happen to me would be if I gained 25 pounds.
8. I do not have many friends that are fat.
9. Some people are fat because they have no willpower.
10. Although some fat people are surely smart, in general, I think they tend to not be quite as bright as normal weight people.
